# Treating From the Inside Out: Relevance of Fecal Microbiota Transplantation to Counteract Gut Damage in GVHD and HIV Infection

**DOI:** 10.3389/fmed.2020.00421

**Published:** 2020-08-06

**Authors:** Jing Ouyang, Stéphane Isnard, John Lin, Brandon Fombuena, Xiaorong Peng, Seema Nair Parvathy, Yaokai Chen, Michael S. Silverman, Jean-Pierre Routy

**Affiliations:** ^1^Infectious Diseases and Immunity in Global Health Program, McGill University Health Centre, Research Institute, Montreal, QC, Canada; ^2^Chronic Viral Illness Service, McGill University Health Centre, Montreal, QC, Canada; ^3^Chongqing Public Health Medical Center, Chongqing, China; ^4^Department of Microbiology and Immunology, McGill University, Montreal, QC, Canada; ^5^State Key Laboratory for Diagnosis and Treatment of Infectious Diseases, National Clinical Research Center for Infectious Diseases, Collaborative Innovation Center for Diagnosis and Treatment of Infectious Diseases, College of Medicine, The First Affiliated Hospital, Zhejiang University, Hangzhou, China; ^6^Infectious Diseases Division, St. Joseph's Health Care, London, ON, Canada; ^7^Division of Hematology, McGill University Health Centre, Montreal, QC, Canada

**Keywords:** fecal microbiota transplantation, graft-vs.-host disease, HIV infection, gut epithelial damage, dysbiosis

## Abstract

The gastrointestinal (GI) tract is a complex and well-balanced milieu of anatomic and immunological barriers. The epithelial surface of the GI tract is colonized by trillions of microorganisms, known as the gut microbiota, which is considered an “organ” with distinctive endocrine and immunoregulatory functions. Dysregulation of the gut microbiota composition, termed dysbiosis, has been associated with epithelial damage and translocation of microbial products into the circulating blood. Dysbiosis, increased gut permeability and chronic inflammation play a major role on the clinical outcome of inflammatory bowel diseases, graft-vs.-host disease (GVHD) and HIV infection. In this review, we focus on GVHD and HIV infection, conditions sharing gut immune damage leading to dysbiosis. The degree of dysbiosis and level of epithelial gut damage predict poor clinical outcome in both conditions. Emerging interventions are therefore warranted to promote gut microbiota homeostasis and improve intestinal barrier function. Interventions such as anti-inflammatory medications, and probiotics have toxicity and/or limited transitory effects, justifying innovative approaches. Fecal microbiota transplantation (FMT) is one such approach where fecal microorganisms are transferred from healthy donors into the GI tract of the recipient to restore microbiota composition in patients with *Clostridium difficile-*induced colitis or inflammatory bowel diseases. Preliminary findings point toward a beneficial effect of FMT to improve GVHD and HIV-related outcomes through the engraftment of beneficial donor bacteria, notably those producing anti-inflammatory metabolites. Herein, we critically review the potential for FMT in alleviating dysbiosis and gut damage in patients with GVHD or HIV-infection. Understanding the underlying mechanism by which FMT restores gut function will pave the way toward novel scalable and targeted interventions.

## Introduction

Trillions of microorganisms reside in the human gut, encompassing not only bacteria but also fungi, archaea, viruses, and eukaryotic microbes, collectively termed microbiota. The gut microbiota was recently considered as an essential organ, playing a critical role in various host functions such as maintenance of the gut barrier and modulation of systemic immune response (1). Furthermore, the endocrine function of the gut microbiota was demonstrated through the production of vitamins and immunoregulatory short chain fatty acids (SCFA) (2). Dysregulation of gut microbiota composition, also known as dysbiosis, can lead to barrier dysfunction and translocation of microbial products leading to systemic inflammation (3). Recent evidence has shown that patients with diabetes, inflammatory bowel diseases (IBD), cancer, graft-vs.-host disease (GVHD) or HIV infection present with gut dysbiosis, gut damage, and microbial translocation (4–7).

Allo-hematopoietic stem cell transplantation (HSCT) is used in the treatment of hematological cancers where donor derived T-cells and natural killer cells target cancer cells in the recipient (4). Occurring after chemotherapy conditioning and HSCT, GVHD may develop as a serious complication when donor immune cells recognize the recipient as foreign and attack healthy cells in host's tissues. GVHD mostly occurs in the gut through the disruption of epithelial tight junctions, destruction of epithelial cells and inflammation in association with dysbiosis (5–8). A large multicenter study showed that gut microbiota composition independently predicted mortality in 1,362 HSCT patients with GVHD (9–11). Similarly, immune damage observed in the gut of people living with HIV (PLWH) was associated with gut dysbiosis, inflammation and clinical outcome (12–15). Despite long-term antiretroviral therapy (ART), damage to the gut mucosa and dysbiosis persist in PLWH, leading to systemic inflammation (8, 10, 11, 15). Like for people with GVHD, PLWH present with a disrupted gut epithelial barrier, immune-mediated intestinal damage, and increased gut permeability (15–22).

Given the association between microbiota composition and clinical outcome in both GVHD and HIV infection (5–11), strategies to modify the gut microbiota have come to light through dietary interventions, the antidiabetic drug metformin, selective antibiotics, probiotics, prebiotics, and fecal microbiota transplantation (FMT) (5, 23, 24). FMT refers to the transfer of fecal microorganisms from healthy donors into the GI tract of patients. It has shown to be effective in *Clostridium difficile* colitis (CDC), IBDs or obesity (25–28). As FMT has been recently shown to improve intestinal barrier function through promotion of gut microbiota homeostasis in GVHD and HIV infection, we discuss its relevance in both conditions in this review (29).

## Dysbiosis and Increased Gut Permeability are Common Features in Patients With GVHD or HIV-Infection

In GVHD or HIV infection, a decrease of gut microbiota diversity is observed and associated with poor clinical outcome (30–33). Compared to patients undergoing allogeneic HSCT without GVHD, patients experiencing GVHD had decreased stool microbial diversity (32). Taur et al. reported that lower bacterial diversity was associated with increased transplant-related mortality in HSCT recipients (33). Nowak et al. also reported that the bacterial diversity of the gut microbiota was correlated with CD4 T-cell counts and inversely correlated with markers of microbial translocation and monocyte activation in PLWH (30).

The gut barrier is organized as a multi-layered and complex system which allows nutrient absorption while preventing the translocation of microbes and their products. Epithelial gut damage occurs in patients with GVHD and PLWH, with damaged enterocytes (basal barrier), non-functional Paneth cells (antimicrobial peptide production) and less mucosal-associated invariant T (MAIT) cells (5, 34–37). Several proteins have been used as gut damage markers. Plasma concentrations of regenerating islet-derived 3-alpha (REG3α), secreted by Paneth cells, were 3-fold higher in patients with gut GVHD at the onset of the disease compared to other HSCT patients (36, 37). Lower levels of REG3α at GVHD onset are correlated with higher 1 year survival (37). In PLWH, we observed that REG3α but not intestinal fatty acid binding protein (I-FABP) plasma levels were correlated with HIV disease progression, microbial translocation and immune activation (36). Similarly, soluble suppression of tumorigenicity (sST2) was also used to predict gut damage and clinical outcomes in patients with GVHD and PLWH (38–42).

Epithelial gut damage allows microbial translocation of microbial products from the lumen to the bloodstream, inducing local and systemic inflammation (43). Circulating levels of lipopolysaccharide (LPS), a pro-inflammatory bacterial cell wall component, is a clinically significant marker to assess the level of microbial translocation (44). LPS leakage in the circulation could induce innate immune activation, in association with mortality in GVHD (45–47). In PLWH, we and others have shown that LPS translocation is correlated with immune dysfunction and increased risk of non-AIDS comorbidities (48–51). Additionally, cytomegalovirus (CMV) primarily replicates in mucosal epithelial cells, decreasing gut barrier integrity. In patients with GVHD and PLWH, CMV latent infection or reactivation is associated with poor clinical outcomes (52–57). These findings suggest that patients with GVHD and HIV infection share similar features in gut damage and related microbial translocation.

Moreover, the gut microbiota can influence host cell physiology via production of metabolites such as SCFAs and bile acids. SCFAs, especially butyrate, constitute the primary energy source for colon epithelial cells. SCFAs play an important role in protecting intestinal barrier function, preventing microbial translocation and reducing inflammation through regulation of host epigenetics (58–60). GVHD patients or PLWH present with a lower abundance of SCFA-producing bacteria and a lower level of SCFAs, compared to non-GVHD HSCT patients or HIV-negative individuals, respectively (61–64). In both conditions, lower levels of SCFAs have been associated with gut damage and inflammation (62, 64–66). Conflicting results exist on the role of butyrate in GVHD as one report shows that patients developing GVHD had higher butyrate production (67). Furthermore, microbiota modulation leading to poor bile acids reabsorption could also be associated with gut damage in both patients with GVHD or PLWH (68–72).

Globally, gut dysbiosis, increased gut permeability, inflammation and systemic immune activation are common features of patients with GVHD or PLWH.

## FMT in Patients With Gut GVHD

Given the dysbiosis and gut permeability in patients with GVHD, and regarding the vital role of gut microbiota in intestinal barrier and homeostasis, strategies targeting the microbiota offer one promising avenue for preventing or treating this condition. In the 1990s, investigators attempted to prevent the development of acute GVHD by drastically reducing the gut microbiota mass with antibiotics, removing the triggers of inflammation (73–75). However, newer studies have proven that gut microbiota-depleted patients had a higher risk of developing acute GVHD following HSCT than non-depleted patients (76, 77). Therefore, strategies promoting a “healthy” microbiota including FMT have attracted recent attention. Kakihana et al. (78) conducted a pilot study on four patients with steroid-resistant or steroid-dependent gut GVHD to observe the effects of FMT from spouses or relatives via nasoduodenal tube. All patients responded to FMT with three complete responses, one partial response, all in absence of severe adverse events. Spindelboeck et al. (79) reported successful FMT in three patients with severe acute GVHD. After one to six FMTs delivered via colonoscopy, all three patients showed increased diversity of the gut microbiota, with two complete remissions of GVHD and one partial remission. Qi et al. (80) reported eight patients with steroid-refractory gut GVHD receiving FMT through a nasoduodenal tube, from a stool bank. After FMT, all patients' clinical symptoms were relieved, bacteria diversity was enriched, and the gut microbiota diversity was restored. Compared to those who did not receive FMT, these eight patients achieved a longer progression-free survival. These case studies suggest that FMT can serve as a promising therapeutic option for gut GVHD, however larger controlled studies are required to confirm these effects.

## FMT in PLWH

In PLWH, the mucosal immune system is disturbed by HIV infection. Th17 and Th22 cells, important components of mucosal immunity, are rapidly depleted following HIV or simian immunodeficiency virus (SIV) infection, contributing to a reduced barrier integrity, microbial translocation, and systemic immune activation (81–83). In a pilot study, Hensley-McBain et al. (84) reported that FMT significantly increases the number of peripheral Th17 and Th22 cells and reduced CD4 T-cell activation in the gut in SIV-infected macaques receiving ART. Moreover, the transplant was well-tolerated and no side effects were observed (84).

**Figure 1:**
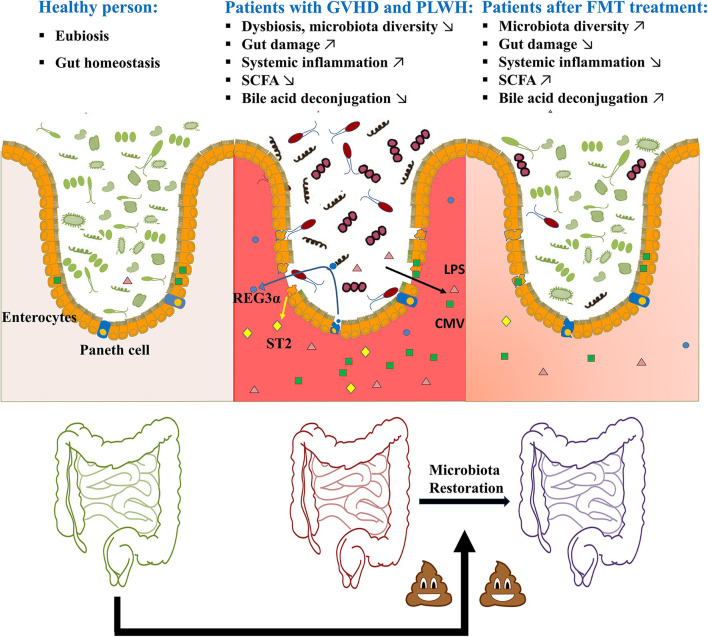
Potential of FMT in GVHD or HIV infection-related gut damage.

A pilot study in ART-suppressed individuals who received one-time FMT from stool bank via colonoscopy reported no serious adverse effects during the 24 weeks of follow-up. Microbial engraftment occurred but was partial, and limited to specific bacterial taxa including an increase of Faecalibacterium (85), which has been shown to exert anti-inflammatory effects in murine experimental colitis (86, 87). The authors considered that the limited effects of FMT might be related to the single dose of FMT given and the absence of antibiotic pre-treatment to “provide space” before FMT (85). Serrano-Villar et al. reported that repeated oral capsular FMT was one way to safely introduce incremental compositional changes into the gut microbiota in ART-treated PLWH (88). Compared to placebo, FMT significantly decreased the gut damage marker I-FABP 4 weeks after initiating FMT. Furthermore, mild engraftment of the donor's microbiota persisted until week 36 after initiating FMT and greater engraftment was observed among the four subjects who had received antibiotics in the 12 week period before FMT (88) (Figure 1).

Safety should be the primary focus of any intervention. Concern persists on the safety of FMT administration, even more so in immunosuppressed recipients. However, PLWH with low CD4 T-cell count were shown to have the most profound modification of their gut microbiota and therefore would benefit greatly from FMT (89). As reviewed by Shogbesan et al. (27), FMT is successful in the treatment of recurrent CDC in immunocompromised patients including organ transplant recipients and PLWH. Encouragingly, FMT showed similar rates of adverse events in immunocompromised participants compared to immunocompetent ones including PLWH with CD4 counts lower than 200 cells/mm^3^ (90–93). Additionally, Schunemann et al. showed that FMT increased CD4 counts in an individual with HIV (94). To better assess the efficacy and safety of FMT, well-designed RCT clinical trials are ongoing and presented in Table 1. However, large studies assessing the influence of FMT in PLWH are still needed.

**Table 1:** Ongoing clinical trials using FMT as a treatment for GVHD and HIV.

**Condition and Aim**	**Design**	**Intervention**	**Number of participants**	**Country**	**Clinical trial number**
**Patients with GVHD**					
GVHD prevention	RCT	ARM I: total gut decontamination + FMT via enema ARM II: FMT via enema Arm III: standard therapy	120	US	NCT03862079
GVHD prevention	RCT	ARM I: Oral FMT Capsule ARM II: Oral placebo Capsule	120	US	NCT03678493
GVHD prevention	RCT	ARM I: FMT capsules ARM II: placebo capsules	48	US	NCT03720392
Acute GVHD treatment	Single arm	Autologous FMT via nasogastric tube	70	Israel	NCT03492502
Steroid refractory acute GI GVHD treatment	Single arm	FMT	32	France	NCT03359980
Acute GI GVHD treatment	Single arm	FMT under colonoscopy or gastroscopy	30	China	NCT03812705
Refractory GVHD treatment	Single arm	FMT via nasojejunal tube	15	China	NCT03549676
Acute GVHD treatment	Single arm	FMT instilled into caecum or terminal ileum	15	Austria	NCT03819803
Gut acute GVHD treatment	Single arm	Oral FMT capsules	4	Israel	NCT03214289
Severe acute gut GVHD treatment	Single arm	Oral FMT capsules	20	US	NCT04280471
Severe acute intestinal GVHD treatment	Single arm	FMT capsules +ruxolitinib+steroids	20	Russia	NCT04269850
GI acute GVHD treatment	Single arm	Oral FMT capsules	17	US	NCT04059757
High-risk acute GVHD treatment	Single arm	Oral FMT capsules	11	US	NCT04139577
Steroid resistant gut acute GVHD treatment	Single arm	FMT via colonoscopy or duodenal tube	30	China	NCT04285424
**PLWH**					
HIV infections treatment	RCT	ARM I: FMT capsules and ART ARM II: placebo capsules and ART	22	Mexico	NCT04165200
Safety of FMT in PLWH	Single arm	FMT capsules	6	US	NCT03329560
Microbiota restoration in PLWH	RCT	ARM I: FMT capsules ARM II: Placebo capsules	30	Spain	NCT03008941

*FMT, Fecal microbiota transplantation; GVHD, Graft-vs.-host disease; PLWH, People living with HIV; ART, Antiretroviral therapy; RCT, Randomized Controlled Trial*.

## Challenges of FMT for Patients With GVHD and PLWH

FMT needs further confirmation of its efficacy in decreasing gut damage in patient with GVHD or PLWH since studies assessing FMT with GVHD or PLWH involved a small number of participants. Moreover, safety needs to be validated as rare side effects may not have been observed in small studies. Therefore, challenges in designing formulations, preventing potential risks and implementing application in clinic for patients with GVHD and PLWH still remain.

Firstly, both healthy donors and patients have a microbiota composition with a high inter-person variability, and the key factors causing microbiota composition variation over time are not fully characterized. The precise influence of different microbiota composition and metabolites on epithelial barrier and clinical outcomes remain poorly understood and need further studies to define their distinctive role on the development of GVHD and HIV infection. Therefore, it remains difficult to select donors and special products for FMT formulation. Moreover, FMT treatment may carry pathogens for digestive and bloodstream infection, as DeFilipp et al. recently reported two cases of drug-resistant *Escherichia coli* bacteremia transmitted by FMT (95). Therefore, despite the absence of a uniform standard for “qualified” microbial communities, donors have to be thoroughly screened for transmissible diseases (e.g., HIV and hepatitis) and other non-infectious conditions (e.g., obesity and diabetes) that may be influenced by changes in the microbiome. In the light of Coronavirus Disease 2019, efforts to screen for novel infectious diseases should be implemented in the future. SARS-CoV-2, the virus that causes this disease, was found in stools even after diminution of respiratory symptoms and could be transmitted through a fecal-oral route (96, 97). Donors who may transfer undesirable agents (e.g., antibiotics, anti-acid proton pump inhibitors, systemic immunosuppressive agents, antineoplastic agents, and glucocorticoids) which can affect the safety or efficacy of FMT should also be excluded (98). Hence, screening for potential donors is costly and time consuming (99). Fortunately, new techniques allow freezing and storage of donor stools for extended periods of time, possibly facilitating FMT implementation (100).

As donor selection is a difficult process, and in order to favor clinical improvement, engraftment of the donor's microbiota should be optimal. Antibiotic conditioning given to the recipient just before FMT seems to improve microbiota engraftment (88). This procedure may destabilize the existing microbial community and promote engraftment of another community. By preventing niche competition in the mucosa between the xenomicrobiota and indigenous microbiota, preparing the gut with antibiotics was shown to facilitate xenomicrobiota colonization, thus enhancing the overall gut microbiota modification efficiency (101). Preliminary results by Serrano-villar et al. showed greater engraftment in four PLWH who had received antibiotics before FMT (88). Pre-therapy with antibiotics before FMT to alleviate GVHD is currently under study (NCT03862079, Table 1).

Encouragingly, multiple clinical trials studying the potential of FMT as a treatment for GVHD or HIV-related gut damage are ongoing (Table 1). In these trials, several routes of administration for FMT are under investigations, including oral capsules, nasal tube, colonoscopy, or enema. The optimal administration route may depend on the characteristics of the disease, and general condition of the patient. Compared with enema, colonoscopy could deliver the FMT to deep cecum, and increase engraftment while the donor stools are expelled less rapidly. However, colonoscopy remains a relatively invasive procedure (102): Kelly et al. reported one case of death from lung-aspiration injury during sedation for FMT administered via colonoscopy (103). Furthermore, nasal administration is considered inconvenient as some cases of intestinal bleeding and rare peritonitis have been reported (104). However, oral capsules have been developed to pass through the acidic environment of the stomach and ensure a delayed delivery of live microbial communities into the intestine (105). By using questionnaires, this route is considered to be most convenient for patients. Kao et al. compared oral capsule and colonoscopy delivered FMT on recurrent CDC showed similar efficacy, with less adverse events (106). Further studies should analyze the preferential route of FMT to alleviate gut damage patients in GVHD and PLWH.

## Conclusion

Both gut GVHD and HIV infection have been associated with dysbiosis and increased gut permeability, contributing to microbial translocation, inflammation, and poor clinical outcomes. Progress has been made in discerning the role of the microbiota in GVHD patients and PLWH. Manipulating the gut microbiota with FMT has been successfully used to treat CDC through microbiota restoration and has paved the way as a novel strategy to improve the outcomes of GVHD patients and PLWH. Several clinical trials are ongoing to assess the efficacy and safety of treating GVHD and HIV-induced gut damage with FMT. However, most trials and published studies are pilot or case series, thus making it difficult to confirm its efficacy and safety. Only large multicentre RCT studies will address the merit of such intervention. Moreover, a standard FMT procedure needs to be implemented and described, including pre-treatment with antibiotics and delivery with oral capsules to favor engraftment. Overall, collaborative efforts encompassing microbiology, clinical care, and pharmacy will define the optimal procedure and number of FMT to obtain a significant and lasting benefit from FMT for individuals with GVHD and HIV. In the future, FMT will pave avenues toward the characterization of important species and their metabolites in modulation of gut damage in patient with GVHD or PLWH, leading to more effective interventions.

## Author Contributions

JO and SI wrote the first draft of the manuscript. JL, BF, XP, SN, YC, and MS provided critical revision of the manuscript. J-PR conceived and designed the manuscript. All authors approved it for publication.

## Conflict of Interest

The authors declare that the research was conducted in the absence of any commercial or financial relationships that could be construed as a potential conflict of interest.
